# Successful Treatment of Drug-Resistant Seizures Secondary to Ring 20 Mosaicism with Perampanel as an Add-On Antiepileptic Drug

**DOI:** 10.1155/2022/7414628

**Published:** 2022-05-26

**Authors:** Janet Ling, Wai Lan Yeung, Kam Lun Hon, Ivan F. M. Lo, Ho-Ming Luk, Cheuk Wing Fung, Alexander K. C. Leung

**Affiliations:** ^1^The Hong Kong Children's Hospital, Kowloon Bay, Hong Kong; ^2^Clinical Genetic Service, Department of Health, HKSAR, Kowloon Bay, Hong Kong; ^3^Clinical Genetics Service Unit, Hong Kong Children's Hospital, Ngau Tau Kok, Hong Kong; ^4^Department of Pediatrics, The University of Calgary, Alberta Children's Hospital, Calgary, Alberta, Canada

## Abstract

We report a girl with drug-resistant seizures, progressive behavioral changes, and cognitive decline. Investigations showed abnormal EEG with frequent high-voltage bifrontotemporal sharp and slow waves, especially during sleep. Seizures were difficult to control, despite the usage of various antiepileptic drugs. Perampanel as an add-on antiepileptic drug appeared efficacious. Due to the recognizable pattern of seizures and EEG findings, a karyotype study was performed which revealed 46 chromosomes with a ring 20 chromosome mosaicism. Ring 20 chromosome is associated with drug-resistant refractory seizures, cognitive decline, and behavioral problems. This case highlights the difficulty and challenge faced in managing drug-resistant refractory seizures associated with ring 20 chromosome. While ring 20 chromosome is often underdiagnosed, one should have a high index of awareness and suspicion of such rare epilepsy syndrome, so that an early diagnosis can be made.

## 1. Introduction

Intractable seizures in children pose diagnostic challenges to the paediatricians [1]. They are also difficult to manage. Ring chromosome 20 (r(20)) syndrome is a rare, often underdiagnosed, and mostly sporadic disease, with just over 150 cases reported globally thus far [2–4]. Rings formation is due to intrachromosomal fusions, albeit the underlying mechanism is poorly understood [5]. We describe a 7-year-old Chinese girl with intractable seizures secondary to r(20) mosaicism who responded to perampanel as an add-on antiepileptic drug. Familiarity of the clinical features and EEG findings of r(20) leads to an early diagnosis through karyotyping.

## 2. Case Report

A 7-year-old Chinese girl born to nonconsanguineous healthy parents presented with refractory seizures. The perinatal history was unremarkable. There was no family history of seizures. Her parents reported that she was a slow learner since early childhood. At the age of six years, she started to have visual hallucinations on awakening including seeing ghosts and insects with clouding of consciousness. Parents thought that they were night terrors. Then, she developed daytime blanking episodes, which subsequently evolved to eye staring, confusion, frightened expression, and stiffening of limbs. Each episode lasted approximately 10 minutes with a frequency of about twice a week. Medical consultation was sought, and she was diagnosed to have focal seizures with impaired awareness and secondary generalisation. Levetiracetam and sodium valproate were started. However, the seizures were not under controlled with frequent nocturnal clustering, up to 10 times a night and semiology changed to jerking of all limbs that resolved spontaneously after approximately 3 minutes. Over the course of 3 months, she also demonstrated significant academic deterioration and behavioral changes.

**Figure 1:**
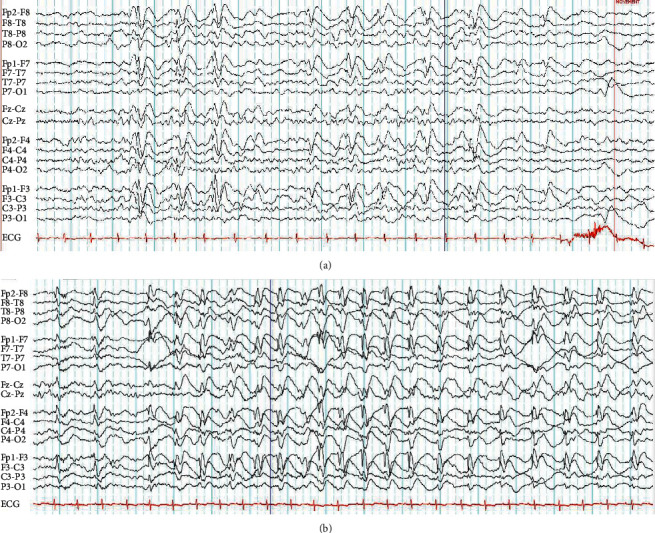
EEG during drowsy state with frequent intermittent high-voltage sharp and slow waves predominantly over bilateral frontopolar and frontotemporal regions (a) and EEG during sleep with long lasting and nearly continuous high-voltage sharp and slow waves over same regions (b). Sensitivity of both EEG tracings is 15 *u*V/mm.

Series of EEG showed frequent high-voltage bifrontotemporal and frontopolar sharp and slow waves which became more diffuse and frequent during sleep (Figure 1). MRI of the brain, metabolic work-up, and autoimmune work-up were unremarkable.

**Figure 2:**
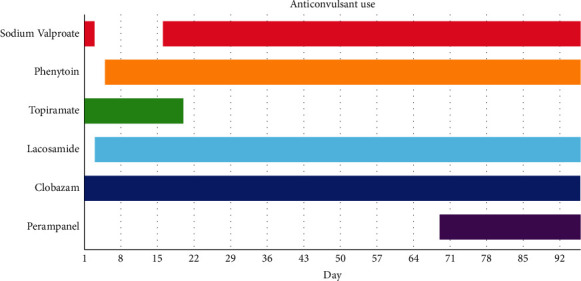
Timeline of various anticonvulsant use.

**Figure 3:**
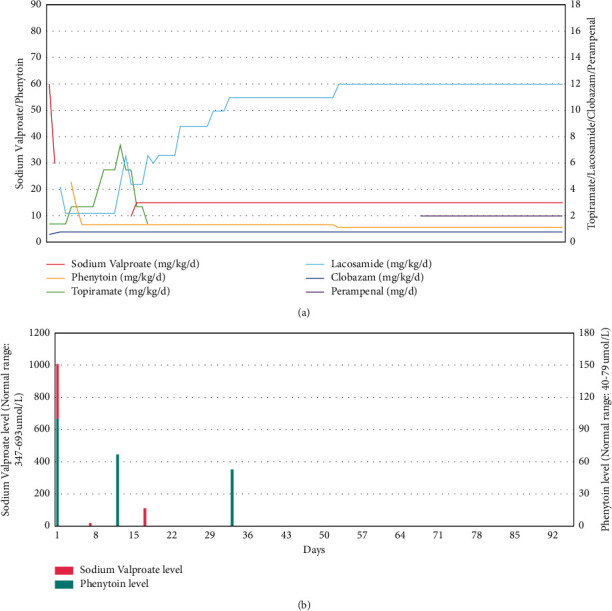
(a) Individual drug dosage over time. Sodium valproate (Epilim) and phenytoin dosage in mg/kg/d on the left. Topiramate, lacosamide, clobazam dosage, and perampanel level in mg/kg on the right. (b) Serum drug level over time. Sodium valproate (umol/L) in red. Phenytoin level (umol/L) in blue.

In the meantime, the seizure clustering became more frequent. She was suspected to have electrical status epilepticus in sleep. In one episode, it was severe enough to require intensive care monitoring and management. The seizure semiology was observed to be absence-like or focal with limb jerking or asymmetrical tonic seizures with fluctuating consciousness, lasting less than 1 minute and worse at night. Clinically, she had no focal neurological signs and no obvious dysmorphic features except bushy eyebrows. She was put on a combination of sodium valproate (loading 40 mg/kg + maintenance 20 mg/kg/d), clobazam (0.8 mg/kg/d), and topiramate (1.4 mg/kg/d), but she developed hyperammonaemia with ammonia level up to 384* μ*·mmol/L, necessitating rescue treatment with sodium benzoate (Figure 2). Her encephalopathic state then improved upon stopping of sodium valproate. Her seizures were partially improved but remained drug-resistant despite courses of intravenous immunoglobulin, high-dose steroids, and other antiepileptic drugs including lacosamide and phenytoin (Figures 2 and 3). Her seizures were not completely aborted, but with frequency down to 2-3 times a day, each episode lasting less than 20 seconds.

**Figure 4:**
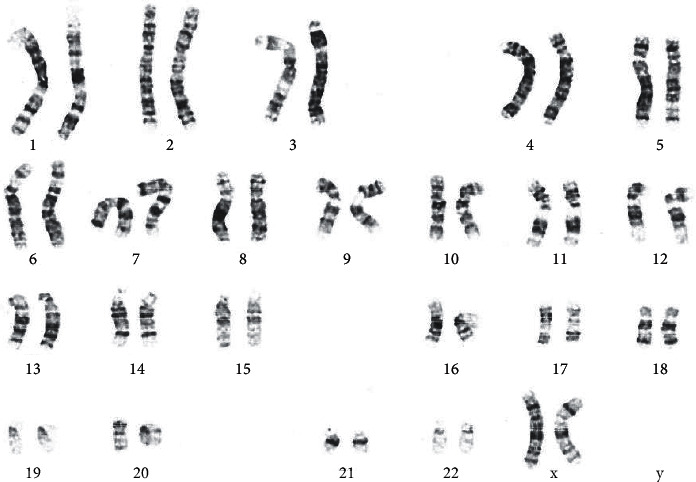
Karyotype of the patient showing 46,XX,r (20)(p13q13.3)/46,XX with mosaicism of 30%.

The diagnosis of r(20) was highly suspected by a neurologist in view of specific electroclinical epilepsy pattern of this girl. Urgent karyotyping with the G-banding technique was performed on cultured peripheral blood lymphocytes at 400–550 band resolution level, with a total of 50 cells counted (Figure 4). The result showed 46,XX,r(20)(p13q13.3) [15]/46,XX [35], which means a mosaic female karyotype with one normal and one abnormal cell lines at the band level of 400–550 resolution was detected. The abnormal cell line had a complete ring formation in chromosome 20 which constitutes 15/50 (30%) cells counted in this peripheral blood sample. Chromosomal microarray showed no copy number gain or loss particularly at chromosome 20, confirming the diagnosis of mosaic ring 20 chromosome.

The girl's seizures were under control after perampanel was added on to the existing treatment with lacosamide, clobazam, and valproate (Figures 2 and 3). Currently, she only had brief eye staring episodes for 10–20 seconds, 2-3 times a week. She was assessed by a clinical psychologist 3 months after discharge from hospital. Her IQ was found to be 77 by Wechsler Intelligence Scale for Children Hong Kong (WISC-HK) (i.e., borderline intellectual functioning). The child had limited range of cognitive ability, mild impairment in visual memory functioning, and poor in abstract verbal reasoning. She required rehabilitation and education support.

When followed up at 1 year, she had about 3 bouts of seizures a week that were mostly brief focal seizures, with impaired awareness lasting for 10–20 seconds and occasionally up to 1-2 minutes. Sometimes, she would be seizure free for 5–7 days. Her IQ assessment by WISC-HK was 77 (i.e., borderline intellectual functioning) 3 months after discharge and 62 (extremely low range) 2 years after discharge, with well-preserved general adaptive functioning. Her seizure control remained stable in terms of long-term efficacy with perampanel.

## 3. Discussion

The r(20) syndrome is a recognizable electroclinical epilepsy syndrome that is characterised by seizure onset usually in childhood with frequent daytime complex partial seizures and nocturnal tonic seizures with an EEG pattern with long periods of bilateral high amplitude slow activity and intermixed spikes. Seizure control is usually difficult and associated with significant comorbidities such as intellectual disability, developmental regression, and dysmorphism.

The uniqueness of this case is the clinical feature of initial terrifying hallucination and absence-like seizures, later evolving to refractory nocturnal frontal lobe seizures, with asymmetric tonic limbs and eye deviations, and typical EEG findings of prominent frequent high-voltage bifrontotemporal epileptiform discharges which lead us to look for the rare r(20) syndrome as a cause of child's drug-resistant seizures [2, 6]. There are two subtypes of r(20) syndrome, namely, mosaic and nonmosaic [7]. Seizures usually start around 7–9 years in patients with mosaic r(20) syndrome and 2.5 years in patients with nonmosaic r(20) syndrome. There is, however, no significant difference in the seizure frequency between two subtypes [8]. The proposed pathophysiologic mechanism for r(20) syndrome include dynamic ring instability, gene dosage change at the telomeric region, and positional silencing that affect the gene expression. However, the exact mechanism remains to be elucidated.

The r(20) syndrome is characterised by an electroclinical triad of drug-resistant refractory frontal seizures, usually worse at night; recurrent nonconvulsive status epilepticus (NCSE); and typical EEG findings of brief frontal epileptic discharges and long lasting high-voltage slow waves with occasional unilateral or bilateral frontal spikes [4]. The r(20) syndrome is associated with progressive deterioration in cognition and behavior in a previously normal child [9]. Variable degrees of mental retardation, dysmorphism, and primary growth failure have also been reported [8, 10]. Higher percentage of r(20) chromosome cells are related to an earlier onset of seizures and is thought to be associated with more severe intellectual impairment but not with the response to drug treatment [2, 8, 11].

Nocturnal frontal seizures and recurrent nonconvulsive status epilepticus, although characteristic, are not diagnostic of r(20) syndrome. In addition, the EEG findings of high amplitude spike/sharp and slow wave at both frontal and temporal regions though characteristic of r(20) syndrome can also be seen in other types of seizures [7]. No major structural abnormalities relating to r(20) syndrome have been reported in the past [7]. The diagnosis of this syndrome can only be achieved by karyotyping. As two-thirds of r(20) cases are mosaic and have no associated deletion and duplication, the diagnosis may be missed by the array-based technique or even whole exome sequencing [8].

Seizures seen in patients with r(20) syndrome are notorious for being drug-resistant to treatment with antiepileptic drugs [1]. Cognitive decline and behavioral deviations could have been aggravated by AEDs such as topiramate and levetiracetam.

In our patient, the conscious state improved upon treatment with a cocktail of antiepileptic drugs, but focal/absence-like seizures remained, although improved in frequency and duration. It was also difficult to find the balance between her tolerance and the side effects, as she developed hyperammonaemia related to valproate in combination with topiramate; the side effects associated with this kind of treatment have been reported before [12]. Ketogenic diet and vagus nerve stimulation have been reported in literature with limited success, which further increases the challenge in the management of seizures seen in patients with r(20) syndrome [1, 6, 13]. Perampanel, an orally active, selective, noncompetitive *α*-amino-3-hydroxy-5-methyl-4-isoxazolepropionic acid receptor antagonist, is an antiepileptic drug that requires once-daily administration [14]. It has been shown that add-on perampanel ≤12 mg/day significantly improves seizure control in patients aged ≥12 years who are experiencing drug-resistant seizures despite ongoing treatment with stable dosages of one to three antiepileptic drugs. The present report suggests that perampanel can be used with success in patients at a younger age. Adjunctive perampanel therapy is generally well tolerated. Adverse events are most commonly CNS-related (e.g., dizziness, somnolence, fatigue, and irritability) and dose-related. Most adverse events are of mild to moderate intensity. Perampanel appears useful as an add-on antiepileptic drug for our patient with r(20) syndrome. For those children with drug-resistant seizures secondary to r(20) syndrome who fail to respond to conventional antiepileptic drugs, we suggest that perampanel be considered as an add-on antiepileptic drug.

We performed a PubMed search using the keywords “Ring chromosome 20” and “perampanel” and had no hit. Hence, we believe this is the first report of the use of perampanel to treat a patient with r(20) syndrome.

Perampanel may have the characteristics for being a wide spectrum antiseizure medication [15]. A systematic review show one randomised controlled trial provided class I evidence of the efficacy and tolerability of adjunctive perampanel for primary generalised tonic-clonic seizures in patients aged ≥12 years with idiopathic generalised epilepsies. Other studies provide weaker observational evidence of its effectiveness in multiple generalised seizure types, including myoclonic, absence, and tonic seizures. [15]

Cannabidiol (CBD) has antiseizure properties, but with no euphoric or intrusive side effects. [16] A systematic review identified patients of both pediatric and adult age, and purified CBD was administered at dosages up to 50 mg/kg/day [16]. The effectiveness of CBD is in the treatment of children and adults presenting with other epilepsy syndromes including CDKL5 deficiency disorder and Aicardi, Dup15q, and Doose syndromes, SYNGAP1 encephalopathy, and epilepsy with myoclonic absences. The most common adverse events observed during treatment with CBD included somnolence, decreased appetite, diarrhea, and increased serum aminotransferases and conclude response to treatment with CBD can be seen in patients across a broad range of epilepsy disorders and etiologies. It is speculated that CBD could be considered in r(20) syndrome.

## 4. Conclusion

We present an interesting and challenge case of r(20) syndrome with drug-resistant refractory seizures, cognitive decline, and behavioral problems. The drug-resistant seizures responded to perampanel when used as an add-on epileptic drug. This case also highlights the difficulty and challenge faced in the diagnosis and management of drug-resistant seizures associated with r(20) syndrome. While r(20) syndrome is often underdiagnosed, there should have a high index of awareness and suspicion, so that an early diagnosis can be made with karyotyping [6, 13].

## Data Availability

The data used to support this study are included within the article.
